# Anemoside B4 targets RAGE to attenuate ferroptosis in sepsis-induced acute lung injury

**DOI:** 10.3389/fphar.2025.1590797

**Published:** 2025-07-31

**Authors:** Yue Bu, Zhixi Li, Cheng Wang, Yongjing Yu, Chang Liu, Yue Sun, Zhenyu Sun, Weidong Gong, Juan Luo, Ziyong Yue

**Affiliations:** ^1^ Department of Anesthesiology, Second Affiliated Hospital of Harbin Medical University, Harbin, China; ^2^ Heilongjiang Province Key Laboratory of Research on Anesthesiology and Critical Care Medicine, Harbin, China; ^3^ Department of Pain Medicine, Second Affiliated Hospital of Harbin Medical University, Harbin, China; ^4^ The Key Laboratory of Myocardial Ischemia Organization, Chinese Ministry of Education, Harbin, China; ^5^ Department of Enviromental Hygiene, School of Public Health, Harbin Medical University, Harbin, China; ^6^ Department of Continuing Education, Second Affiliated Hospital of Harbin Medical University, Harbin, China

**Keywords:** anemoside B4, sepsis, acute lung injury, ferroptosis, network pharmacology, AGE/RAGE pathway

## Abstract

**Introduction:**

Anemoside B4 (AB4), a major bioactive saponin isolated from the roots of *Pulsatilla chinensis*, exhibits anti-inflammatory and antioxidant properties. While recent study has demonstrated its ability to inhibit ferroptosis in arthritis, its role in sepsis-induced acute lung injury (SALI) remains undefined. This study aims to clarify the mechanism underlying AB4’s action in SALI.

**Methods:**

To explore the therapeutic mechanism of AB4 in SALI, an integrated approach was employed, combining network pharmacology, molecular dynamics simulation, surface plasmon resonance (SPR) assays, *in vivo* experiments using cecal ligation and puncture (CLP)-induced mouse models, and *in vitro* studies with lipopolysaccharide (LPS)-stimulated RAW264.7 cells. The therapeutic effects of AB4 on SALI were evaluated through histopathological examination, biochemical analysis, immunofluorescence staining, and Western blotting, which collectively elucidated its *in vivo* and *in vitro* mechanisms of action.

**Results:**

AB4 ameliorated CLP-induced lung injury in mice, as evidenced by reduced pathological damage, lower injury scores, and a decreased lung wet-to-dry weight ratio. *In vivo*, AB4 significantly reduced levels of pro-inflammatory cytokines (IL-1β, TNF-α, and IL-6), decreased oxidative stress markers MDA and DHE, and increased GSH levels. *In vitro*, AB4 inhibited ferroptosis in macrophages, with a pharmacological effect comparable to the known ferroptosis inhibitor Ferrostatin-1 (Fer-1). Network pharmacology analysis identified the AGE/RAGE signaling pathway as a primary target of AB4. AB4 dose-dependently downregulated RAGE expression and restored levels of GPX4 and SLC7A11. SPR and molecular docking experiments confirmed a high affinity between AB4 and RAGE, with a dissociation constant (KD) of 3.86 μM. Consistently, co-administration of the RAGE inhibitor FPS-ZM1 effectively suppressed ferroptosis and enhanced Nrf2 activity in CLP-induced mice.

**Conclusion:**

AB4 directly targets RAGE to inhibit the AGE/RAGE-Nrf2 axis, thereby suppressing both ferroptosis and inflammation in SALI. This previously unreported mechanism establishes AB4 as a novel multifaceted therapeutic candidate for SALI.

## 1 Introduction

The latest definition of sepsis describes it as life-threatening organ dysfunction caused by an imbalance in the host’s response to infection (Zhang and Ning, 2021). SALI is one of the most common and severe complications of sepsis. The pathogenesis of SALI is multifaceted, with accumulating evidence highlighting the pivotal role of pulmonary inflammation in disease progression (He et al., 2023). Alveolar macrophages drive the initiation and perpetuation of pulmonary inflammatory responses by secreting excessive pro-inflammatory cytokines, including tumor necrosis factor-α (TNF-α) and interleukins (IL-6, IL-1β) (Malainou et al., 2023). Thus, the discovery of effective therapeutic agents remains a pressing need to advance SALI treatment strategies. Ferroptosis, an iron-dependent regulated cell death driven by lipid peroxidation, contributes to SALI (Li et al., 2018). Emerging evidence indicates that ferroptosis plays an important role in the pathogenesis of sepsis (Wu et al., 2024; Shen et al., 2023) and is often the downstream result of redox imbalance (Dong et al., 2021; Li et al., 2022; Shen et al., 2023). Inhibiting macrophage ferroptosis plays a protective role in SALI (Lai et al., 2023).

Pulsatilla chinensis (Bunge) Regel [Ranunculaceae] (validated via http://www.plantsoftheworldonline.org) is a biotanical drug used in traditional Chinese medicine (TCM), primarily for fever and dysentery due to its heat-clearing, detoxifying, and blood-cooling properties. Anemoside B4 (AB4), an isolated major purified triterpenoid saponin constituting>4.6% of *P. chinensis* roots (Li et al., 2020; Jin et al., 2018), exhibits multifaceted pharmacological activities, including antimicrobial, antiviral, antitumor, and anti-inflammatory effects (He et al., 2019; Kang et al., 2019; Li et al., 2019). Notably, its anti-inflammatory and immunomodulatory properties have been well characterized in numerous studies, with consistent evidence supporting its pharmacological effects in mitigating inflammatory responses (Kang et al., 2019; He et al., 2020; Li et al., 2020; Ma et al., 2022; Cao et al., 2023; Shen et al., 2023). However, its protective pharmacological effects on SALI remain poorly defined; critically, no studies have explored AB4’s regulatory role in ferroptosis—a key driver of SALI pathogenesis (He et al., 2022)—nor its mechanistic association with SALI. Therefore, this study investigates the protective effects of AB4 on SALI and its underlying mechanisms both *in vitro* and *in vivo*, with a focus on ferroptosis-related pathological processes.

The AGE/RAGE signaling pathway is a recognized master regulator of sepsis-induced organ injury (Lutterloh et al., 2007). The AGE/RAGE interactions amplify oxidative stress and inflammation, disrupt iron homeostasis, and indirectly promote ferroptosis (Chen et al., 2024). However, whether AB4 directly targets the AGE/RAGE signaling pathway—and how this might suppress ferroptosis in SALI—remains entirely unexplored. While a recent study showed that AB4 alleviates arthritis via ferroptosis suppression (Guo et al., 2024), this mechanism remains unverified in SALI. Thus, AB4’s targeting of the AGE/RAGE-ferroptosis axis in SALI represents a fundamental knowledge gap.

**FIGURE 1:**
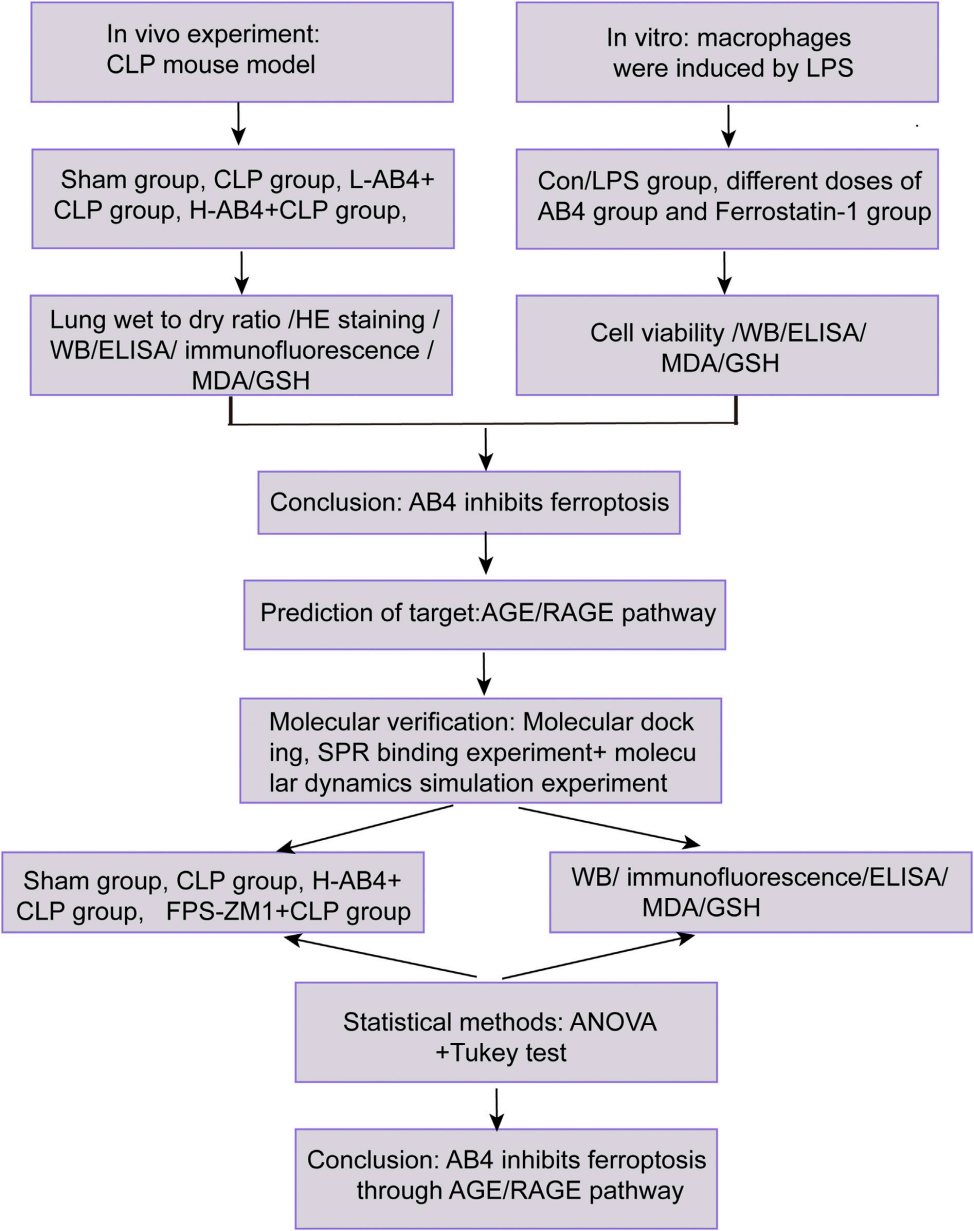
Experimental workflow for evaluating AB4 in SALI. Schematic overview of *in vivo* (CLP mouse model) and *in vitro* (LPS-stimulated macrophages) studies, target prediction (AGE/RAGE signaling pathway), and molecular verification.

To address this gap, we employed an integrated strategy (Figure 1): (1) Assess AB4’s inhibition of ferroptosis in SALI models *in vivo* (essential for sepsis-related systemic validation); (2) Identify AB4 targets via network pharmacology; (3) Validate target binding through molecular docking, SPR, and dynamics simulation; (4) Pharmacologically verify ferroptosis-related pathways. This multidisciplinary approach aims to elucidate AB4’s mechanism and advance targeted SALI therapies.

## 2 Methods

### 2.1 Establishment of a sepsis model

Male C57BL/6J mice (*Mus musculus*, NCBI Taxonomy ID: 10090), 8 weeks old with a body weight of 20 ± 2 g, were obtained from the Laboratory Animal Center of Harbin Medical University (Certification:SYXK (Hei) 2024-002). The strain was sourced from Vital River Laboratories (Beijing, China) and corresponds to Jackson Laboratory Stock # 000664. Mice were randomly assigned to five groups (n = 8 per group) and housed under specific pathogen-free (SPF) conditions (temperature: 22°C ± 1°C; humidity: 55% ± 5%; 12-h light/dark cycle) with *ad libitum* access to autoclaved food and water. All animal procedures strictly adhered to the guidelines of the National Institutes of Health (NIH) and were approved by the Institutional Animal Care and Use Committee (IACUC) of Harbin Medical University (Approval No.: SYDW 2023-095). A murine model of sepsis was established using the CLP method as described by He et al. (2020). Briefly, mice were anaesthetised with isoflurane. After making a midline abdominal incision, the cecum was ligated at its distal one-third using 4-0 silk thread and subsequently punctured through the blind end. The incision was closed layer-by-layer. Mice received fluid resuscitation and were kept warm post-surgery.

### 2.2 Drug management

AB4 (HY-N0205, HNMR purity ≥98%, analytical grade) and FPS-ZM1 (HY-19370, LCMS purity ≥ 99.87%) were purchased from MedChemExpress (Monmouth, NJ, USA). A stock solution of AB4 was prepared at a concentration of 50 mg/mL in ddH_2_O, and FPS-ZM1 was prepared as a 30 mM stock solution in DMSO. The effect of AB4 on SALI was investigated using drug dosages and administration times based on previous studies (Kang et al., 2019; Pei and He, 2021; Xie et al., 2024). Two doses of AB4 (50 mg/kg and 100 mg/kg) were used. No positive control group was established, as 50 mg/kg AB4 can reduce sepsis-induced acute liver injury, and its pharmacological effects on pneumonia injury are equivalent to those of dexamethasone (Kang et al., 2019; Pei and He, 2021).

The study comprised five distinct experimental groups (n = 8 per group): (1) Sham operation (Sham group): Mice underwent laparotomy without ligation/puncture; (2) CLP + Vehicle (DMSO) (CLP group): CLP surgery + intraperitoneal (i.p.) DMSO injection immediately post-surgery; (3) CLP + L-AB4 (50 mg/kg) (L-AB4 group): CLP surgery + i.p. AB4 (50 mg/kg) immediately post-surgery; (4) CLP + H-AB4 (100 mg/kg) (H-AB4 group): CLP surgery + i.p. AB4 (100 mg/kg) immediately post-surgery; (5) CLP + FPS-ZM1 (1 mg/kg) group: CLP surgery + i.p. FPS-ZM1 (1 mg/kg) 1 h prior to surgery. All mice were euthanized by cervical dislocation at 24 h post-procedure. Blood samples were collected to measure pro-inflammatory molecule levels. Some lung samples were collected for histopathological examination, and the remaining lung tissues were preserved at −80°C for Western blotting (WB).

### 2.3 Cytotoxic test for drugs

A 96-well plate was seeded with RAW264.7 cells (Chinese Academy of Sciences, Beijing, China) at a density of 5 × 10^3^ cells per well in Dulbecco’s modified Eagle medium (DMEM) supplemented with 10% foetal bovine serum, 100 U/mL penicillin, and streptomycin (Zhang et al., 2023). After 24 h of cultivation, cells were pre-treated with different doses of AB4 (0, 3, 6, 12, 24, 48 and 100 μM) for 4 h, followed by an additional 24 h of cultivation. Cells were treated with a cell counting kit 8 (CCK8; Beyond Biotechnology, Shanghai, China) solution for 1 h. The absorbance at 450 nm was measured using a microplate reader (ELX800, BioTek Instruments Inc., Winooski, VT, USA) to determine cell viability relative to the control group.

### 2.4 Drug cell pharmacological effects determination

After pre-treatment with AB4 (0, 3, 6, 12, 24, 48 and 100 μM) for 4 h, the cells were co-stimulated with 1 μg/mL lipopolysaccharide (LPS) for 24 h. Untreated cells served as the control group; CCK8 solution was added to each well and incubated at 37°C for 1 h. Relative cell viability was calculated by measuring the absorbance at 450 nm and normalizing to the control group (Luo et al., 2022).

### 2.5 Cell culture and processing

RAW264.7 cells were cultivated in high glucose DMEM containing 10% foetal bovine serum and 1% penicillin-streptomycin. Experiments were conducted using cells in the logarithmic growth phase to ensure optimal response. To simulate sepsis, macrophages were treated with 1 μg/mL LPS for 24 h. Based on the results across different concentrations, RAW264.7 cells were assigned to the following groups: 1) control (Con) group: blank group + phosphate-buffered saline (PBS); 2) LPS group: blank group + LPS culture; 3) L-AB4 group: cells cultured with 12 μM AB4 followed by LPS treatment; 4) M-AB4 group: cells cultured with 24 μM AB4 followed by LPS treatment; 5) H-AB4 group: cells cultured with 48 μM AB4 followed by LPS treatment; and 6) Ferrostatin-1 (Fer-1) group: cells pre-treated with 2 μM Fer-1 for 30 min (Matute-Bello et al., 2011) prior to LPS exposure.

### 2.6 Network pharmacology analysis

#### 2.6.1 Identification of potential targets of AB4 and SALI

Potential targets of AB4 were obtained from four databases: Comparative Toxicogenomics (https://ctdbase.org), GeneCards (https://www.genecards.org), SwissTargetPredict (https://www.swisstargetprediction.ch), and TargetNet (https://targetnet.scbdd.com/home/index) databases. All selected data were limited to *Homo sapiens*. The UniProtKB database (https://www.UniProt.org) was utilised to generate official identification numbers and gene symbols for all examined targets. Potential ALI targets were obtained from Comparative Toxicogenomics (https://ctdbase.org), DisGeNET (https://www.disgenet.org), GeneCards (https://www.genecards.org), Online Mendelian Inheritance in Man (OMIM) (https://www.omim.org), and Therapeutic Target (TTD) (https://db.idrblab.net/ttd/) using the search term ‘acute lung injury’. Disease database targets were integrated, and duplicates were removed to obtain genes associated with ALI.

#### 2.6.2 Shared genes among AB4, ferroptosis, and SALI

Ferroptosis-related genes were collected from the FerrDb database (http://www.zhounan.org/ferrdb/) and supplemented with newly identified ferroptosis-associated genes from recent studies (Fang et al., 2023; Chen et al., 2023; Huang et al., 2022). A Venn diagram was used to intersect AB4 target genes, ferroptosis-related genes, and SALI-related genes, with the overlapping genes representing common targets among the three.

#### 2.6.3 GO and KEGG enrichment analyses

Gene Ontology (GO) and Kyoto Encyclopedia of Genes and Genomes (KEGG) enrichment analyses of the intersecting genes were performed using the “clusterProfiler” package in R software. Significant enrichment results were visualized using the ggplot2 package.

#### 2.6.4 Molecular docking

The crystal structure of the Receptor for Advanced Glycation End Products (RAGE) protein was retrieved from the PDB database (PDB ID: 6xq1) and preprocessed as follows: (1) removal of water molecules, ions, and native ligands using PyMOL 2.5.2; (2) addition of polar hydrogens and assignment of AMBER ff14SB charges via PDB2PQR 3.5.1; and (3) energy minimization with 5,000 steps of steepest descent in GROMACS 2022.3 (force constant = 1,000 kJ/mol/nm^2^) under the MMFF94 force field. The binding site was defined as residues within 10 Å of the co-crystallized ligand.

Molecular docking was performed using AutoDock Vina 1.2.3. Prior to docking, the receptor protein was further processed in PyMOL 2.5.2 to remove residual water molecules, salt ions, and small molecules. A docking box (25 × 25 × 25 Å^3^) was centered at the centroid of the native ligand in the crystal structure. All processed small molecules and receptor proteins were converted to the PDBQT format required for docking using ADFRSuite 1.03. The global search parameter was set to 32, and default settings were used for other parameters. The highest-scoring docking conformation was selected as the optimal binding mode, and results were visualized and analyzed using PyMOL 2.5.2.

#### 2.6.5 Surface plasmon resonance (SPR)

The binding affinity between AB4 and RAGE protein was measured using Surface Plasmon Resonance (SPR). Recombinant RAGE protein was immobilized on a CM5 chip (#BR-1005-30) via carboxyl groups on the dextran matrix. After immobilization, various concentrations of AB4 were injected over the protein-coupled chip surface. Binding assays and data analysis were performed using a Biacore T200 system.

#### 2.6.6 Molecular dynamics simulations

Based on the docking results, the small molecule-protein complex was used as the initial structure for full-atom molecular dynamics simulations using AMBER 22. Prior to simulation, the charge of AB4 was calculated using the antechamber module in AMBER 22 and the Hartree-Fock (HF) SCF/6-31G* basis set in Gaussian 09. The small molecule (AB4) was parameterized using the GAFF2 force field, and the protein (RAGE) was assigned parameters using the ff14SB force field. For each system, hydrogen atoms were added using the LEaP module, and a truncated octahedral TIP3P water box was applied with a 10 Å buffer from the solute. Na^+^ and Cl^−^ ions were added to neutralize the system charge. Finally, topology and parameter files for the simulation were generated, and molecular dynamics simulations were performed using AMBER 22. Prior to the formal simulations, energy minimization was carried out through 2,500 steps of the steepest descent method followed by 2,500 steps of the conjugate gradient method. After energy minimization, the system was gradually heated from 0 to 298.15 K at a constant volume and heating rate over 200 ps. Subsequently, the system was maintained at 298.15 K, and a 500 ps NVT (isothermal-isochoric) ensemble simulation was performed to achieve uniform distribution of solvent molecules within the solvent box. Finally, a 500 ps equilibrium simulation was conducted under NPT (isothermal-isobaric) conditions. The composite system was then subjected to a 100 ns NPT (isothermal-isobaric) simulation under periodic boundary conditions. In the simulations, the cutoff distance for non-bonded interactions was set to 10 Å. Long-range electrostatic interactions were calculated using the Particle Mesh Ewald (PME) method, and the SHAKE algorithm was applied to constrain the bond lengths involving hydrogen atoms. The system pressure was maintained at 1 atm, with an integration time step of 2 fs, and trajectories were saved every 10 ps for subsequent analyses.

#### 2.6.7 MM/GBSA-based binding free energy calculation

The binding free energies between the protein and ligand in all systems were calculated using the MM/GBSA (molecular mechanics/generalized Born surface area) method. In this study, the trajectory from 90 to 100 ns was used for the calculations, with the specific formula as follows:

$${\Delta G}_{bind}={\Delta G}_{\text{complex}}\;–\;\left({\Delta G}_{\text{receptor}}+{\Delta G}_{\text{ligand}}\right)$$

$$={\Delta E}_{\text{internal}}+{\Delta E}_{\text{VDW}}+{\Delta E}_{\text{elec}}{+\Delta G}_{\text{GB}}+{\Delta G}_{\text{SA}}$$ (1)

In Equation 1, ${\Delta E}_{\text{internal}}$ represents internal energy, ${\Delta E}_{\text{VDW}}$ represents van der Waals interaction energies, and ${\Delta E}_{\text{elec}}$ represents electrostatic interaction energies. The internal energy includes bond energy (E_bond_), angular energy (E_angle_), and torsional energy (E_torsion_). ${\Delta G}_{\text{GB}}$ and ${\Delta G}_{\text{SA}}$ , collectively referred to as solvation free energy, are the polar and non-polar solvation free energy, respectively. In this study, ${\Delta G}_{\text{GB}}$ was calculated using the GB model developed by Nguyen et al. (*igb* = 2). The non-polar solvation free energy (D*G*_*SA*_) was calculated based on the product of the surface tension (γ) and solvent-accessible surface area (SASA), D*G*_*SA*_ = 0.0072 × DSASA. Entropy change was ignored owing to its high computational resource consumption and low precision (Supplementary Table S3). Simulation reliability was determined by monitoring energy drift, temperature stability, and pressure at a constant temperature of 300 K and pressure of 1 bar to simulate physiological conditions. The following parameters were analysed: average structure calculation, root-mean-square deviation (RMSD), and root-mean-square fluctuation (RMSF).

### 2.7 Enzyme-linked immunosorbent assay (ELISA)

After euthanasia, mouse heart blood was collected, incubated at room temperature (23°C ± 2°C) for 2 h, and centrifuged at 13,500 × *g* for 10 min at 4°C. The supernatant was collected and re-centrifuged at 2,000 × g for 10 min to remove debris, and the final supernatant was retained for subsequent assays. Levels of tumor necrosis factor-α (TNF-α), interleukin-1β (IL-1β), and interleukin-6 (IL-6) were quantified using ELISA kits (Jianglai Biotechnology, Jiangsu, China).

### 2.8 Measurement of glutathione (GSH) and malondialdehyde (MDA) levels

Lung tissues and cells were collected, mixed with 1 mL of extraction buffer, sonicated, homogenized on ice, and centrifuged at 12,000 rgm at 4°C. The supernatant was used to determine GSH (JL-T0906; Jianglai Biotechnology, Jiangsu, China) and MDA (JL-T0761; Jianglai Biotechnology, Jiangsu, China) levels. Absorbance was measured at 412 nm (for GSH) and 532 nm (for MDA) using an Infinite M200 Pro multifunctional microplate reader (Tecan Group Ltd., Mannedorf, Switzerland).

### 2.9 Histopathological assay

The right middle lobe of the lung was rinsed with normal saline, fixed in 4% formalin, embedded in paraffin, and sectioned into 5-μm-thick slices. Sections were deparaffinized and stained with hematoxylin and eosin (H&E). Images were captured using a light microscope. Lung tissue damage was evaluated by two pathologists blinded to the treatment groups, following previously established criteria (Dodson et al., 2019). Image analysis was performed using ImageJ software, an open-source platform for biological image analysis (Schindelin et al., 2012).

### 2.10 Lung wet-to-dry weight ratio

The lung wet-to-dry weight ratio was used to assess pulmonary edema severity (Sun et al., 2022). The left lung was weighed immediately after removing surface moisture to determine the wet weight. Subsequently, the lung tissue was dried in an oven at 65°C for 48 h to measure the dry weight.

### 2.11 Transmission electron microscopy

Lung tissue samples were fixed in glutaraldehyde solution (pH 7.3) at 4°C for 2 h, dehydrated with gradient acetone (50%, 70%, 90%, and 100%), stained with uranyl acetate for 15 min and lead citrate for 10 min, and examined using a transmission electron microscope (HT-7650; Hitachi, Tokyo, Japan).

### 2.12 Dihydroethidium (DHE) staining

DHE staining was used to quantify superoxide anions in lung tissue. Frozen lung sections were thawed, rinsed, and incubated with 10 μM DHE fluorescent probe in the dark at 37°C for 30 min. Tissues were counterstained, washed, and imaged using a fluorescence microscope (EVOS M5000; Thermo Fisher Scientific, USA). Image analysis was performed using ImageJ software.

### 2.13 Immunofluorescence assay

Tissue sections were fixed with 4% paraformaldehyde, permeabilized with 0.3% Triton X-100, and blocked with 5% BSA at 4°C. Sections were incubated with primary antibodies overnight at 4°C, followed by incubation with corresponding secondary antibodies and DAPI staining. Immunofluorescence signals were observed under a confocal microscope (LSM800, LSM 980; Zeiss, Germany) and analyzed using ImageJ software.

### 2.14 Western blotting (WB)

Lung tissue and cells were homogenized on ice for 30 min in RIPA buffer (Shanghai BioTai Bio-Tech Co., Ltd.) containing PMSF (Beyotime Biotechnology, Shanghai, China) at a 100:1 ratio, then centrifuged at 12,000 × g for 20 min at 4°C to collect the supernatant. Protein concentration was determined using the Coomassie brilliant blue method, and 5× loading buffer (dilution ratio 1:4; Shanghai BioTai Bio-Tech Co., Ltd.) was added to the supernatant. Proteins were separated by SDS-PAGE and transferred to PVDF membranes. After blocking, membranes were incubated overnight at 4°C with primary antibodies: anti-PTGS2 (1:3000, YT1073; Affinity, Jiangsu, China), anti-GPX4 (1:10,000, ab125066; Abcam, Cambridge, UK), anti-SLC7A11 (1:10,000, ab175186; Abcam), anti-RAGE (1:3000, ab17247; Abcam), and anti-β-actin (1:200,000, AC026; ABclone, Wuhan, China). Membranes were then incubated with secondary antibodies, and protein bands were detected using ECL reagent (Biosharp, Hefei, China) and analyzed using a BioImage system (Bio-Rad Laboratories, Inc., Hercules, CA, USA) and ImageJ software.

### 2.15 Statistical analysis

All data are expressed as mean ± standard deviation (SD). Statistical analyses were performed using GraphPad Prism 9 software (La Jolla, CA, USA). Significant differences were determined by one-way analysis of variance (ANOVA), with statistical significance set at *p* < 0.05.

## 3 Results

### 3.1 AB4 prevents CLP-induced SALI

**FIGURE 2:**
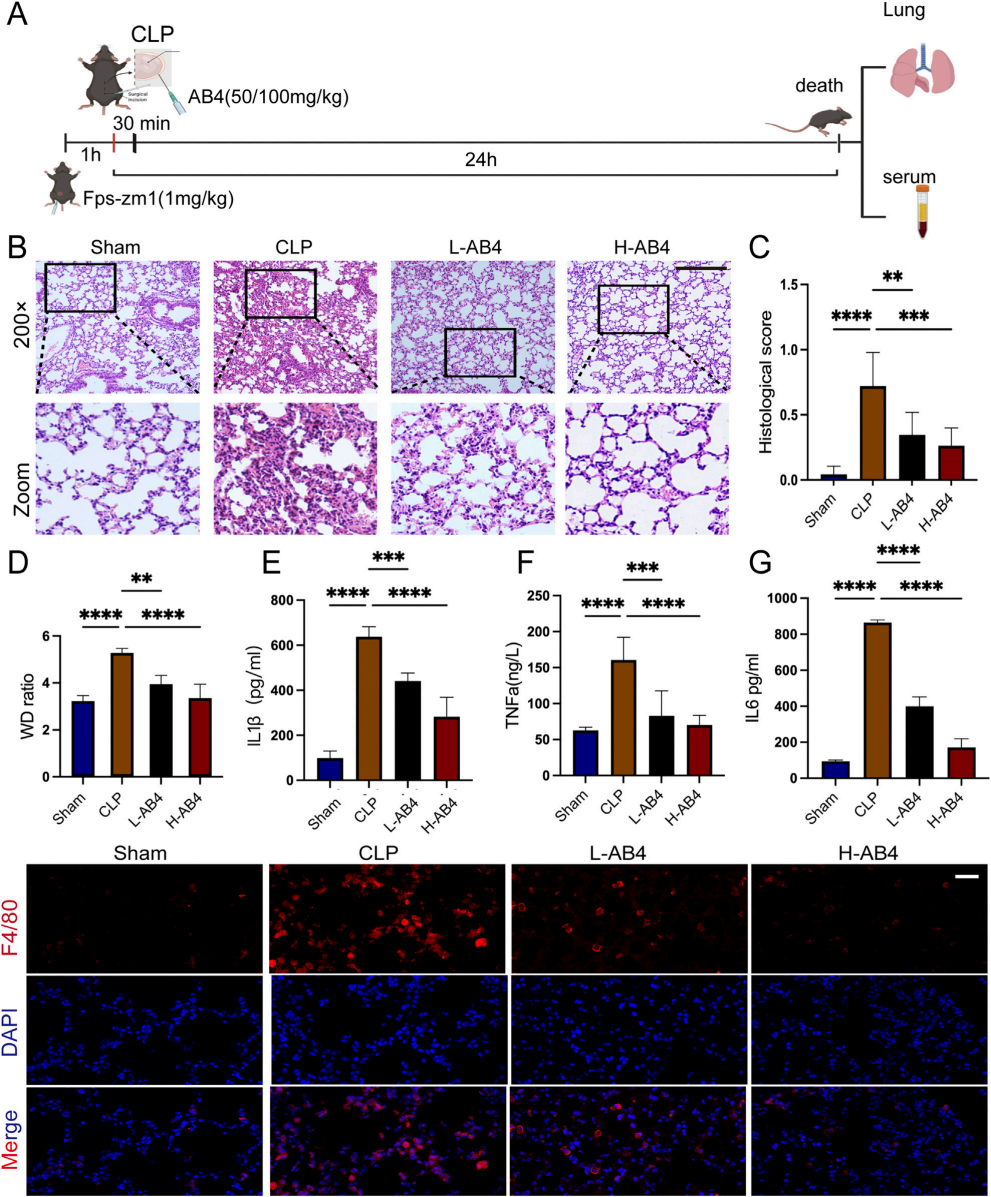
Anemoside B4 (AB4) inhibits inflammation in the lungs of caecal ligation and puncture (CLP) mice. **(A)** Schematic representation of the animal experiment. **(B,C)** Representative H&E staining images and semi-quantitative lung damage scores of lung tissue from each group of mice (magnification ×200, scale bar = 50 μm) (n = 3). **(D)** Determination of the wet/dry weight ratio of lung tissues in each group (n = 5). **(E–G)** The TNF-α, IL-6, and IL-1β levels in mice serum (n = 3). **(H)** Representative images of F4/80 immunohistochemical staining in the lung sections under 400 ×, scale bar = 20 μm. Quantitative data are shown as the mean ± SD, *p < 0.05, **p < 0.01, ***p < 0.001.

Figure 2A illustrates the *in vivo* experimental protocols. Hematoxylin and eosin (H&E) staining of lung tissue from experimental mice revealed thickening of the lung interstitium and increased infiltration of inflammatory cells in the alveolar interstitium and alveolar cavity in the CLP group. In contrast, mice in the AB4 groups, particularly those in the H-AB4 group, exhibited less pronounced pathological alterations (*p* < 0.01) than the CLP group (Figures 2B,C). The lung wet-to-dry weight ratio was significantly higher in the CLP group than in the sham-operated group but decreased significantly (*p* < 0.01) in the AB4 group (Figure 2D). ELISA showed that TNF-α, IL-1β, and IL-6 levels in the CLP group serum were higher than those in the sham group; however, AB4 treatment—especially high-dose AB4—downregulated these inflammatory cytokines (*p* < 0.01; Figures 2E–G). Furthermore, AB4 pretreatment significantly inhibited CLP-induced macrophage infiltration into lung tissue compared with the CLP group (Figure 2H). Collectively, these results suggest that AB4 may inhibit macrophage aggregation to mitigate inflammation and lung tissue damage in CLP-induced sepsis mice.

### 3.2 AB4 alleviates ferroptosis in CLP mice

**FIGURE 3:**
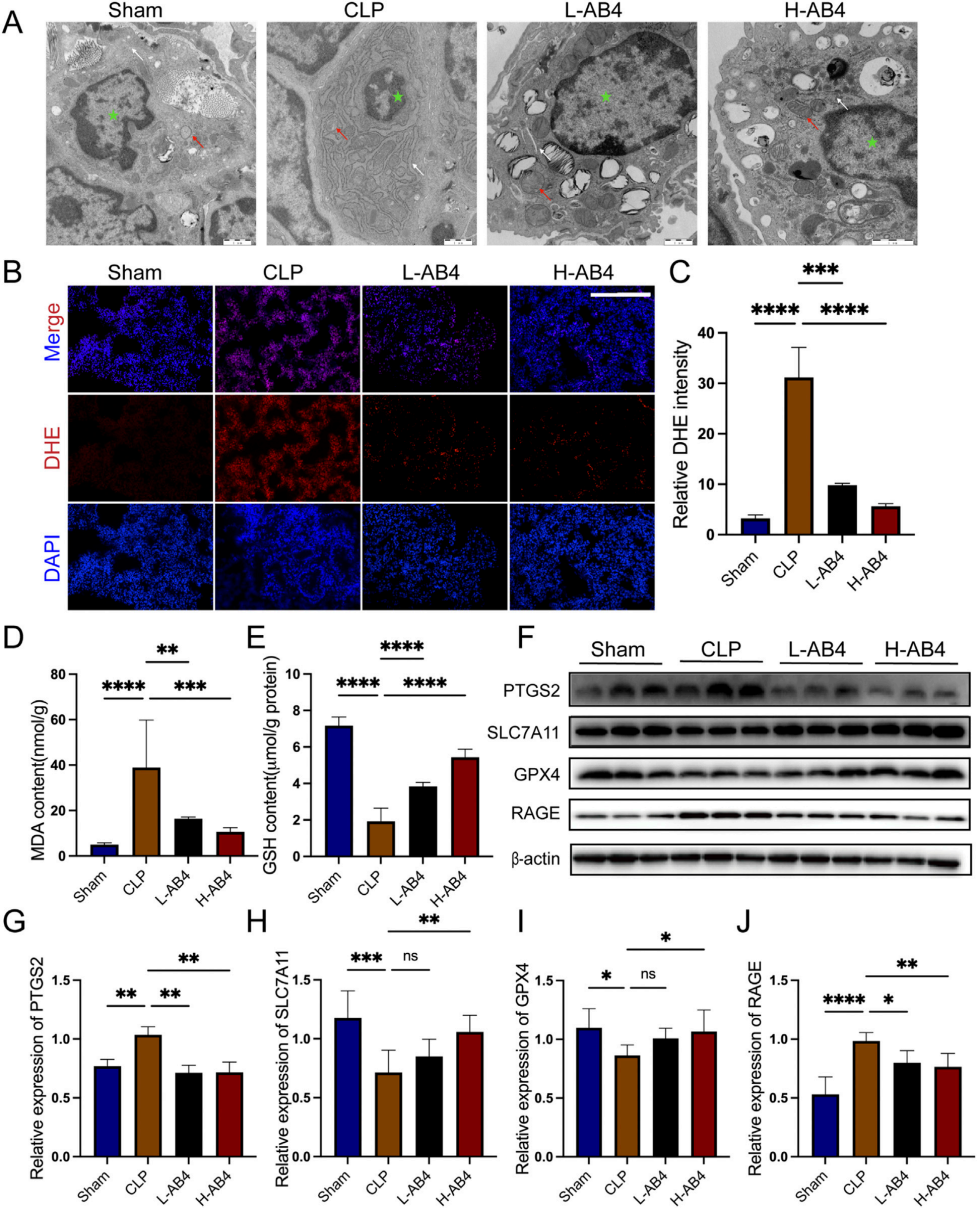
Anemoside B4 (AB4) can reduce ferroptosis. **(A)** Transmission electron microscopy image of lung tissue ultrastructure (×10,000, 1 μm scale). The green star represents the nucleus, the white arrow denotes the endoplasmic reticulum, and the red arrow represents mitochondria. **(B,C)** Images of DHE staining of lungs. Scale bar = 50 μm. Quantitative graph for DHE staining of lungs (n = 3). **(D,E)** The levels of GSH and MDA in the lungs (n = 5). **(F–J)** The protein levels of PTGS2, SLC7A11, RAGE and GPX4 in the lungs (n = 3). Data are shown as the mean ± SD, *p < 0.05, **p < 0.01, ***p < 0.001, ****p < 0.0001, ns, not significant.

Having confirmed the anti-inflammatory pharmacological effects of AB4 in the CLP model, we further explored its pharmacological actions by examining lung tissue changes. In CLP-induced mice, we observed mitochondrial cristae loss and endoplasmic reticulum enlargement—characteristic features of cellular stress. Notably, these mitochondrial damages were significantly mitigated in the H-AB4 treatment group compared with the untreated CLP group (Figure 3A), suggesting that H-AB4 may exert its pharmacological effects by inhibiting ferroptosis. Biochemical analyses revealed that in the CLP model group, the levels of MDA and DHE were significantly elevated, while GSH levels were reduced (all *p* < 0.0001; Figures 3B–E). Conversely, H-AB4 treatment led to a marked decrease in MDA and DHE levels accompanied by increased GSH content (all *p* < 0.0001; Figures 3B–E). We further evaluated the expression of ferroptosis-related genes. In the CLP group, the expression of PTGS2 and RAGE was upregulated, whereas the expression of SLC7A11 and GPX4 was downregulated—changes consistent with the induction of ferroptosis. In contrast, H-AB4 treatment significantly reduced the expression of PTGS2 and RAGE and increased the expression of SLC7A11 and GPX4 (all *p* < 0.05; Figures 3F–J).

### 3.3 *In vitro* determination of effective AB4 concentrations

**FIGURE 4:**
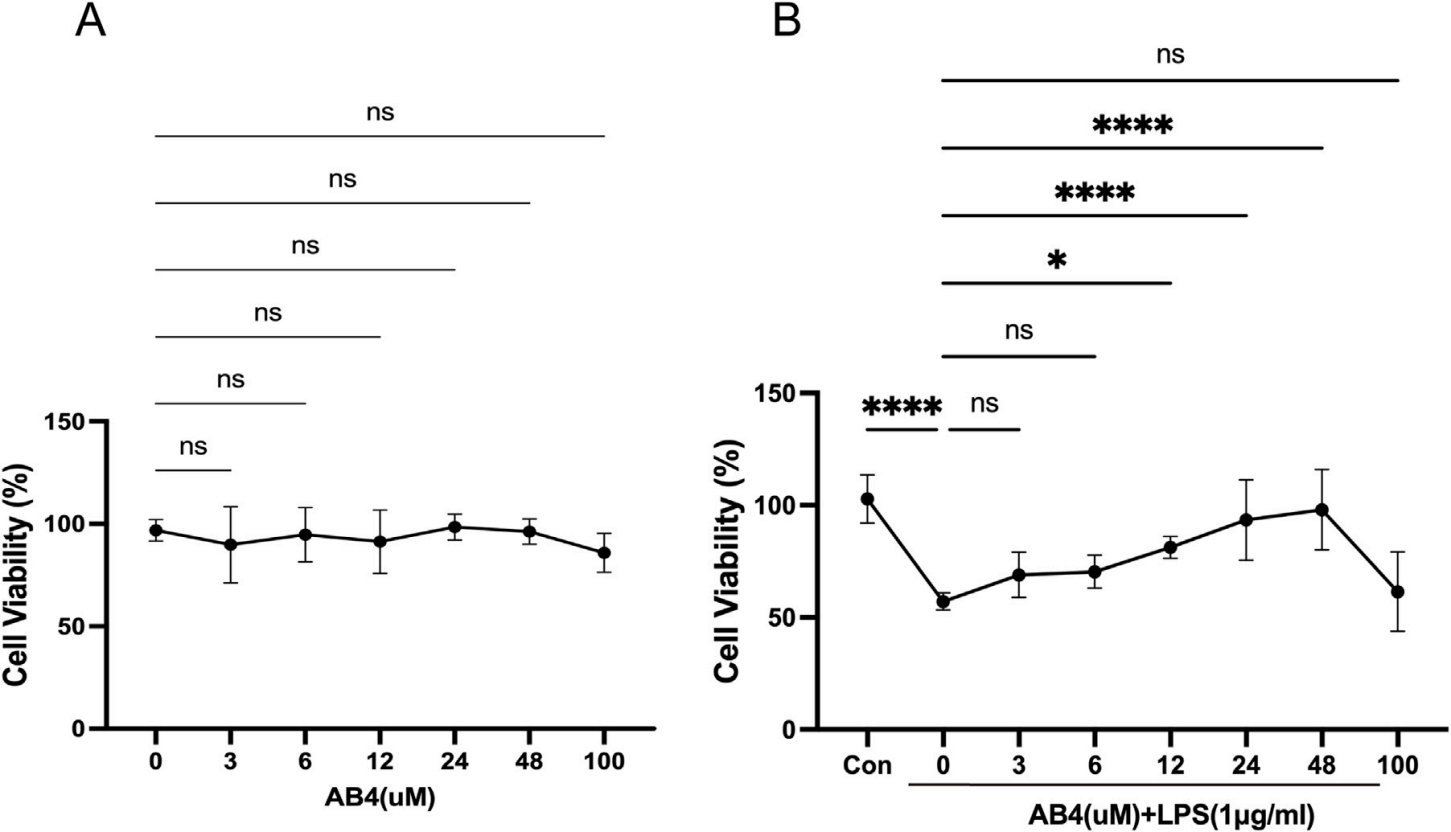
Effects of anemoside B4 (AB4) on RAW264.7 cell viability. **(A)** Assessment of cytotoxic effects of AB4 at different concentrations and LPS (1 μg/mL) on Raw264.7 cells. **(B)** Impact of AB4 on LPS-induced changes in cell viability of Raw264.7 cells. (n = 3) Data are shown as the mean ± SD, *p < 0.05, **p < 0.01, ***p < 0.001, ****p < 0.0001, ns, not significant.

The CCK-8 assay demonstrated that AB4 exerted no toxicity on RAW264.7 cells at concentrations up to 100 μM, with no significant differences in cell viability observed between all AB4-treated groups and the Con group (Figure 4A). Compared with the Con group, the LPS-treated group showed a significant reduction in cell viability (*p* < 0.001). However, pre-treatment with AB4 at concentrations of 12, 24, or 48 μM significantly reversed this LPS-induced viability decrease (*p* < 0.05; Figure 4B). Therefore, these three concentrations were selected for subsequent experiments.

### 3.4 AB4 alleviates ferroptosis in macrophages in a dose-dependent manner

**FIGURE 5:**
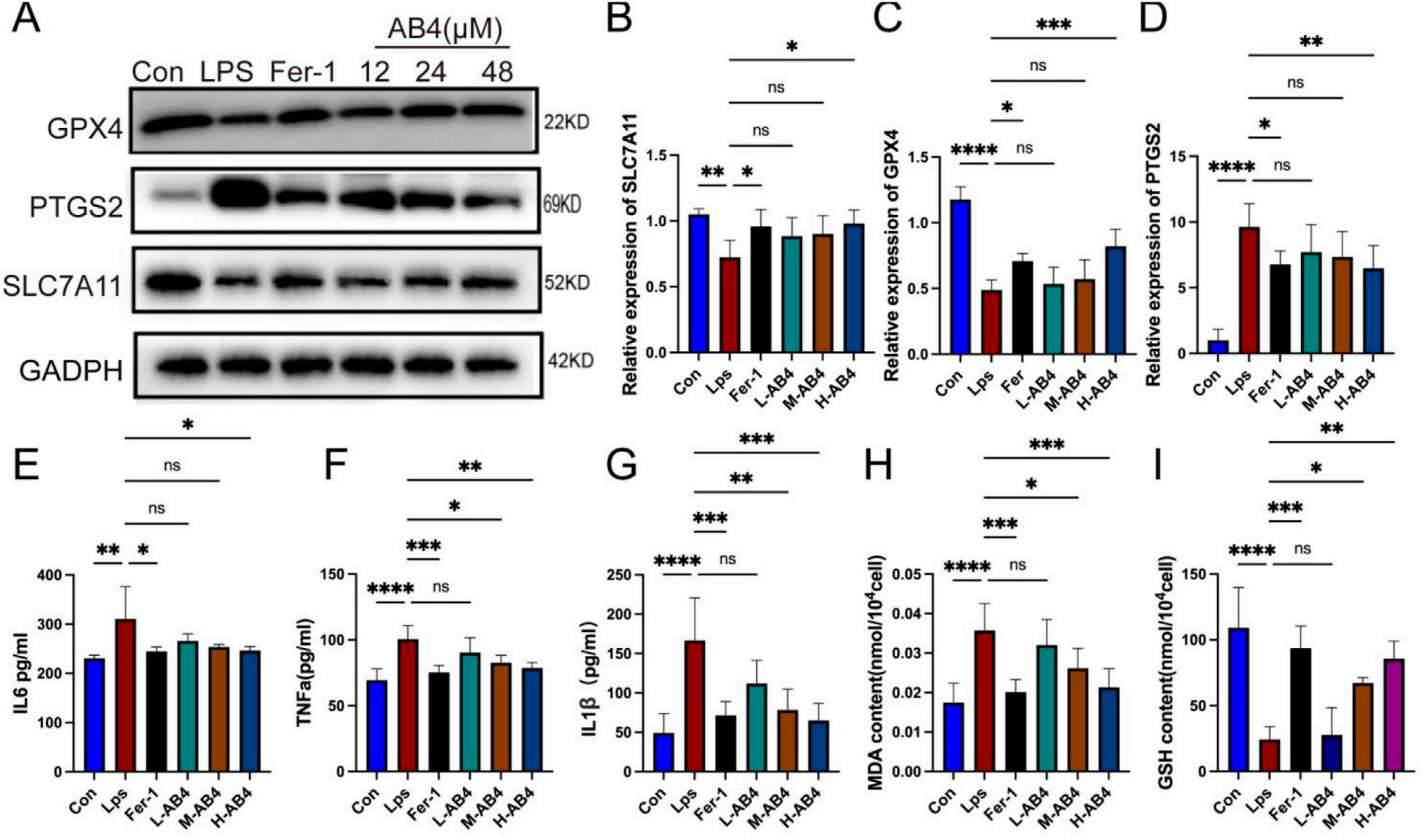
Anemoside B4 (AB4) inhibits ferroptosis of LPS-treated macrophages cells. **(A–D)** Western blot analyses of PTGS2, SLC7A11 and GPX4 (n = 3). **(E–G)** The levels of interleukin (IL) -1β, IL-6, and TNF-α in cell supernatants of each group (n = 3). **(H,I)** The levels of GSH and MDA in the Raw264.7 cells (n = 3). Data are shown as the mean ± SD, *p < 0.05, **p < 0.01, ***p < 0.001, ****p < 0.0001, ns, not significant.

In RAW264.7 macrophages, both the ferroptosis inhibitor Fer-1 and the H-AB4 significantly downregulated the expression of the ferroptosis marker PTGS2, while upregulating the protein levels of GPX4 and SLC7A11 (all *p* < 0.05; Figures 5A–D). Consistent with the *in vivo* results, AB4 reduced the levels of inflammatory factors (TNF-α, IL-1β, and IL-6) in cell supernatants and intracellular MDA levels, while increasing intracellular GSH levels. These findings indicate that AB4 possesses potent anti-ferroptotic and anti-inflammatory properties (all *p* < 0.05; Figures 5E–I). Collectively, these *in vivo* and *in vitro* results suggest that AB4 mitigates lung injury in the CLP-induced mouse model by inhibiting ferroptosis.

### 3.5 Identification and enrichment analysis of AB4-regulated ferroptosis targets in ALI

**FIGURE 6:**
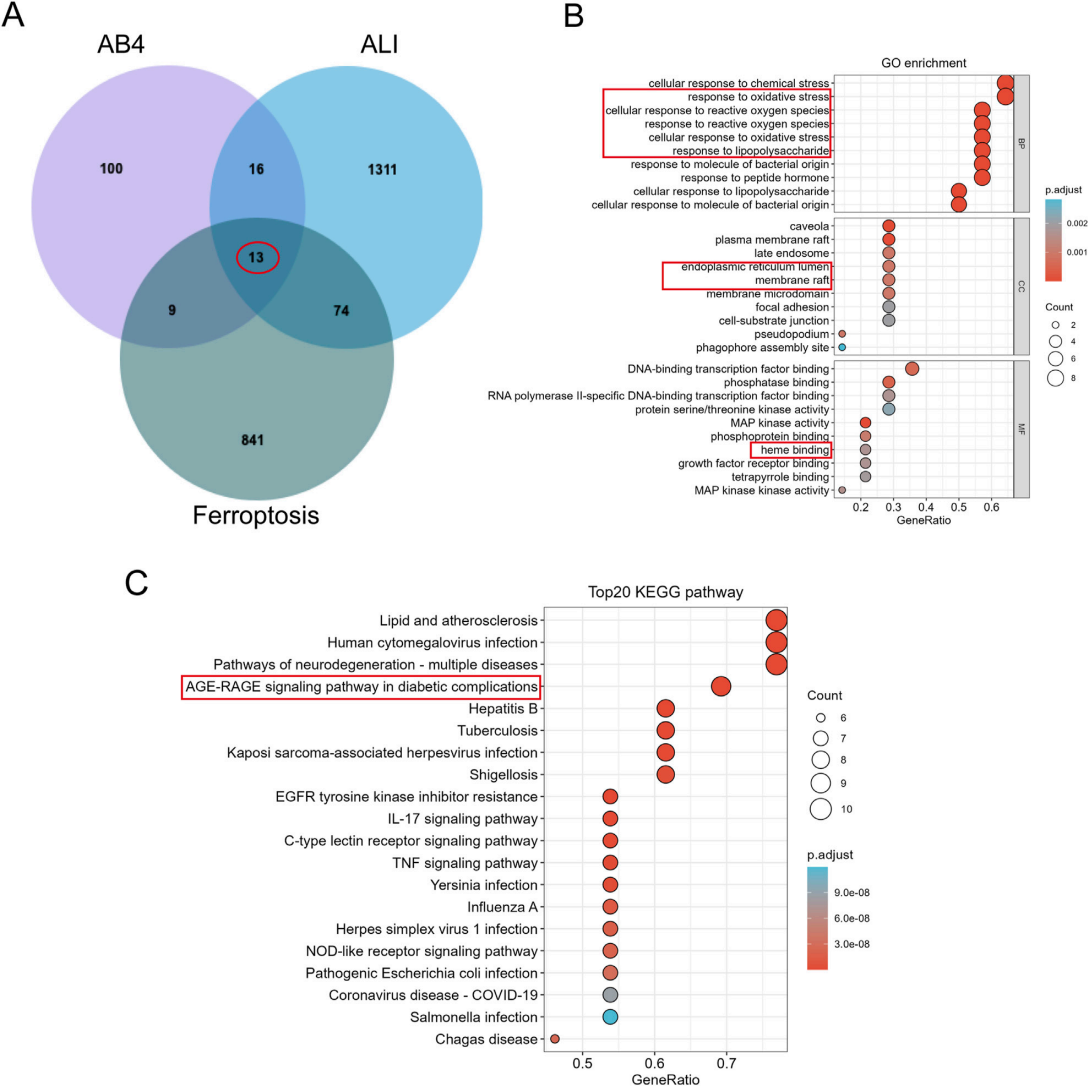
Identification and Enrichment analysis of AB4 regulatory ferroptosis-targets in ALI. **(A)** Venn diagram demonstrating potential targets for AB4-ALI-Ferrdb in the treatment of ALI. **(B)** GO enrichment analysis; **(C)** KEGG enrichment analysis.

To further explore the molecular targets of AB4 in regulating ferroptosis in SALI, we performed network pharmacology analysis. A total of 138 AB4-related genes were retrieved by searching the Comparative Toxicogenomics Database, GeneCards, SwissTargetPrediction (with a probability threshold >0.1), and TargetNet. After removing duplicates, 133 unique target genes were identified (Supplementary Table S1). For disease-related targets, 1,531 genes associated with ALI were obtained from GeneCards (with a relevance score >1), along with 93 genes from DisGeNET, 51 from OMIM, 7 from TargetNet, and 39 from the Comparative Toxicogenomics Database. Following deduplication, 1,414 ALI-related target genes were finalized (Supplementary Table S2). Additionally, 937 ferroptosis-related genes were collected from the FerrDb database, supplemented by newly identified ferroptosis-associated genes from recent studies (Fang et al., 2023; Chen et al., 2023; Huang et al., 2022). Overlap analysis of AB4-related genes, ferroptosis-related genes, and ALI-related genes identified 13 common target genes (Figure 6A).

GO enrichment analysis indicated that these overlapping target proteins are primarily involved in biological processes related to cellular responses to oxidative stress—key events in ferroptosis. In terms of cellular components, the targets are predominantly localized to the endoplasmic reticulum lumen and membrane rafts. For molecular functions, they are mainly associated with heme binding (Figure 6B). KEGG enrichment analysis identified that the common genes are primarily involved in the Advanced Glycation End Products (AGE)/RAGE signaling pathway, IL-17 signaling pathway, and TNF signaling pathway. Among these, the AGE/RAGE signaling pathway was the most significantly enriched, supporting RAGE as a priority target. Collectively, the target intersection analysis suggests that the AGE/RAGE signaling pathway may mediate the therapeutic pharmacological effects of AB4 against SALI, which warrants further experimental validation (Figure 6C).

### 3.6 Molecular docking and SPR analysis

**FIGURE 7:**
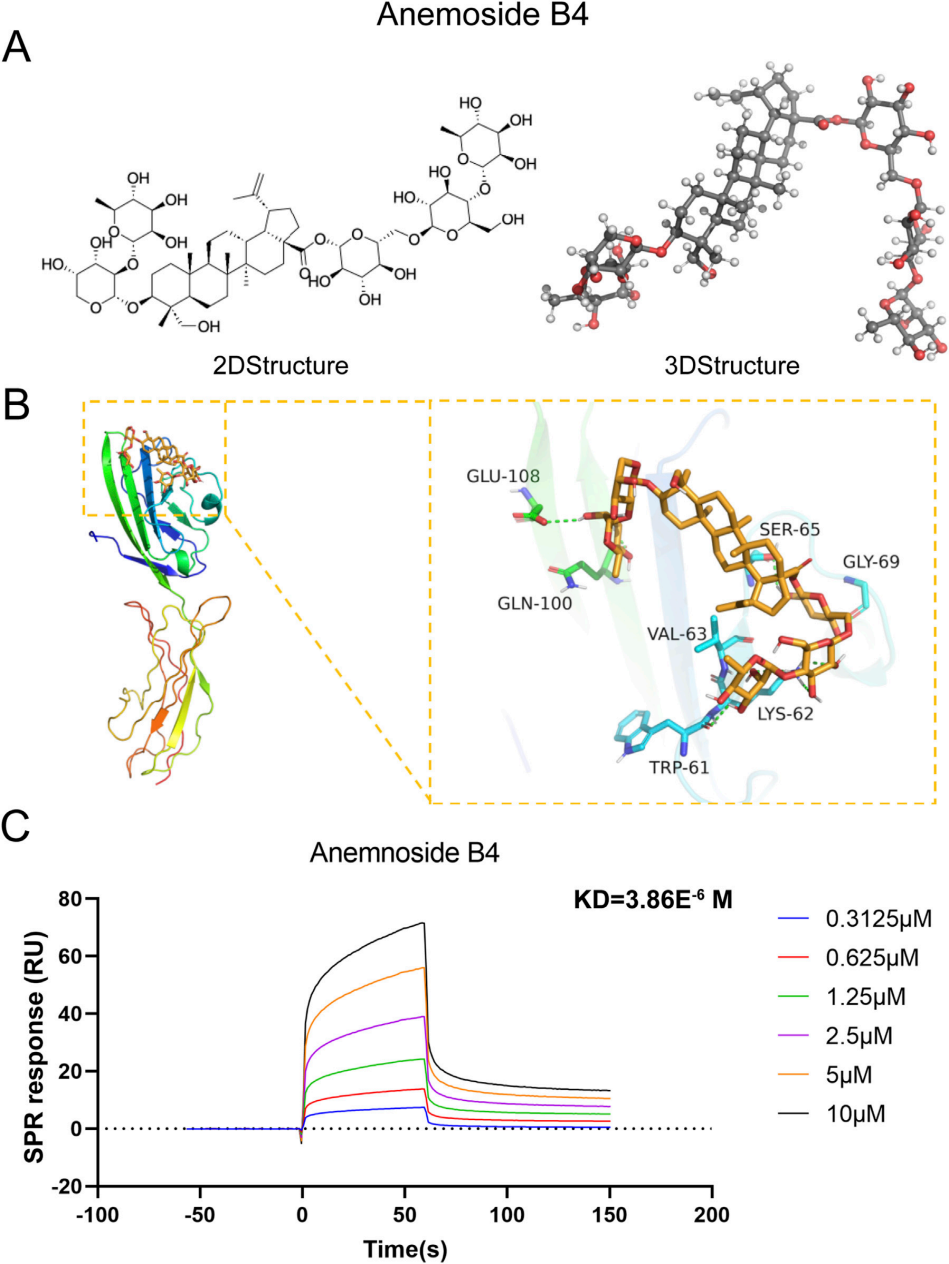
Molecular docking and SPR experiments. **(A)** The two-dimensional and three-dimensional molecular structures and relative molecular weights of AB4. **(B)** Schematic diagram of AB4 and RAGE docking. **(C)** The KD values of the screened ligands were calculated by SPR.

Given that the differentially expressed genes were primarily enriched in the AGE/RAGE signaling pathway, RAGE was hypothesized to be a potential target mediating AB4’s therapeutic pharmacological effects in sepsis. The 2D and 3D molecular structures of AB4 are presented in Figure 7A. Molecular docking simulation is a practical and efficient approach to explore interactions between small molecules and target proteins. Herein, we employed Vina 1.2.3 software to investigate the binding of AB4 to RAGE protein. As shown in Figure 7B, the interaction profile between AB4 and RAGE protein reveals that AB4 forms hydrogen bonds with multiple amino acid residues of RAGE, including GLU-108, GLN-100, SER-65, GLY-69, VAL-63, LYS-62, and TRP-6. These hydrogen bonds enhance the stability of the AB4-RAGE complex. Binding affinity, a key indicator of binding potential (with more negative values indicating stronger binding likelihood), was calculated as −7.152 kcal/mol for the AB4-RAGE interaction, suggesting a favorable binding capacity (Table 1).

**TABLE 1:** Table of molecular docking and efficiency indicators.

Parameter	Value
Docking Software	AutoDock Vina 1.2.3
Grid Box Center (Å)	(12.4, −5.8, 22.1)
Grid Box Size (Å^3^)	25 × 25 × 25
Exhaustiveness	32
Binding Affinity (kcal/mol)	−7.152
Hydrogen Bond Residues	GLU-108, GLN-100, SER-65, GLY-69, VAL-63, LYS-62, TRP-6
Ligand Efficiency (LE)	−7.152/25 = - 0.286
Ligand-Lipophilicity Efficiency (LiE)	pIC50 (−log(10^−7^)) − clogP (2.3) = 4.7

Formula description: “LE = ΔG (kcal/mol)/number of heavy atoms; LiE = −log(IC50) − clogP (clogP calculated by ChemDraw).”

Surface plasmon resonance (SPR) analysis further confirmed the direct binding of AB4 to RAGE, with a moderate affinity (KD = 3.86 μM). Kinetic analysis revealed a fast association rate and slow dissociation rate, resulting in 50% complex persistence at 200 seconds—indicating prolonged target engagement. Additionally, clear concentration-response curves confirmed the absence of non-specific binding (Figure 7C).

### 3.7 Molecular dynamics validation of RAGE-AB4 binding

**FIGURE 8:**
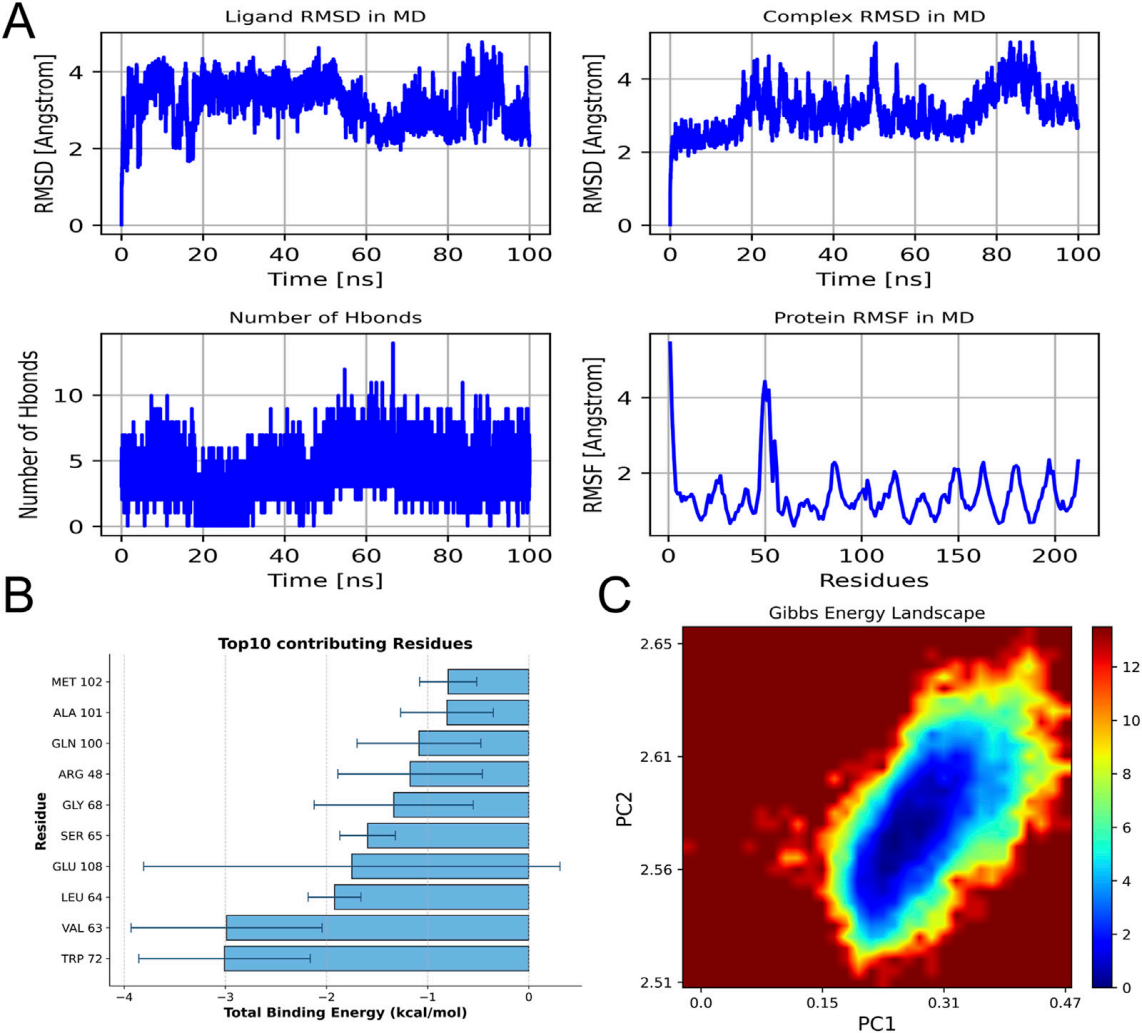
Molecular dynamics simulation. **(A)** RMSD of ligands and ligand protein complexes during simulation; The number of hydrogen bonds between ligands and proteins varies with simulation time, as well as the fluctuation chart of protein RMSF. **(B)** The top 10 amino acids contribute to the binding energy. **(C)** Free energy distribution of protein RAGE and drug AB4 complex.

Molecular dynamics simulations confirmed the stable binding of AB4 to the V-domain of RAGE, with key findings as follows: (1) Structural stability: Over a 100 ns simulation period, the root-mean-square deviation (RMSD) of the ligand (AB4) remained within 2–4 Å, while the RMSD of the RAGE-AB4 complex was maintained at 2–5 Å (Figure 8A). Additionally, a stable number of hydrogen bonds (∼5) and low root-mean-square fluctuation (RMSF) values at the binding site indicated a rigid and stable interaction between AB4 and RAGE. (2) Energetic characteristics: The complex exhibited strong binding affinity, with a calculated MM/GBSA score of −43.41 ± 6.06 kcal/mol (Figure 8B). Energetic analysis revealed that this affinity was primarily driven by van der Waals forces (Supplementary Table S3). Key amino acid residues involved in the interaction included TRP72, VAL63, LEU64, GLU108, and SER65. (3) Conformational stability: Principal component analysis (PCA) showed that the complex predominantly populated low-energy conformational states (Figure 8C), further supporting the stability of AB4-RAGE binding. These molecular dynamics simulations mechanistically validate RAGE as a high-confidence target of AB4, consistent with the prioritization from network pharmacology. The results confirm the stable binding and energetic feasibility of AB4-RAGE interaction, laying a foundation for further experimental investigation.

### 3.8 The AGE/RAGE signaling pathway is critical for AB4-mediated anti-ferroptotic pharmacological effects in SALI

**FIGURE 9:**
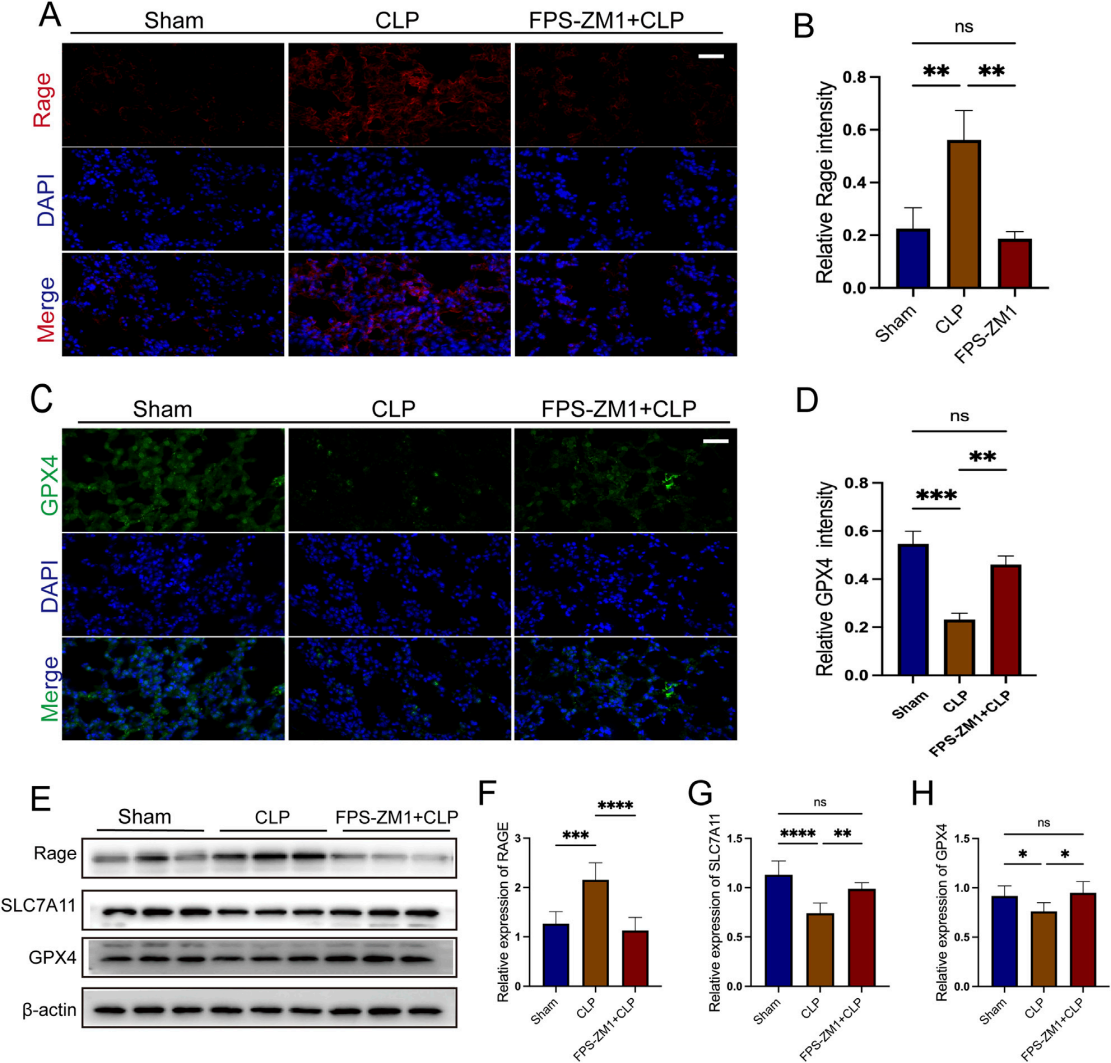
The AGE/RAGE signalling pathway is essential for AB4-mediated anti-ferroptosis in sepsis-associated acute lung injury. **(A,B)** Images of RAGE staining of lungs. Scale bar = 20 μm. Quantitative graph for RAGE staining of lungs (n = 3). **(C,D)** Images of Gpx4 staining of lungs. Scale bar = 20 μm. Quantitative graph for RAGE staining of lungs (n = 3). **(E–H)** The protein levels of SLC7A11, RAGE and GPX4 in the lungs (n = 3). Data are shown as the mean ± SD, *p < 0.05, **p < 0.01, ***p < 0.001, ****p < 0.0001, ns, not significant.

To verify the role of the AGE/RAGE signaling pathway in ferroptosis during SALI, we used FPS-ZM1, a specific inhibitor of RAGE. Compared with the CLP group, FPS-ZM1 treatment markedly attenuated RAGE fluorescence intensity (all *p* < 0.01; Figures 9A,B) and restored GPX4 fluorescence intensity (all *p* < 0.01; Figures 9C,D), indicating concurrent suppression of RAGE signaling and ferroptosis. Consistent with these findings, Western blot analysis revealed that FPS-ZM1-mediated inhibition of RAGE activity ameliorated the CLP-induced downregulation of ferroptosis-related proteins GPX4 and SLC7A11 (all *p* < 0.01; Figures 9E–H). These results confirm that targeting the AGE/RAGE signaling pathway can alleviate ferroptosis in the lung tissue during sepsis.

### 3.9 AB4 inhibits ferroptosis via the RAGE/Nrf2/HO-1 signaling pathway

**FIGURE 10:**
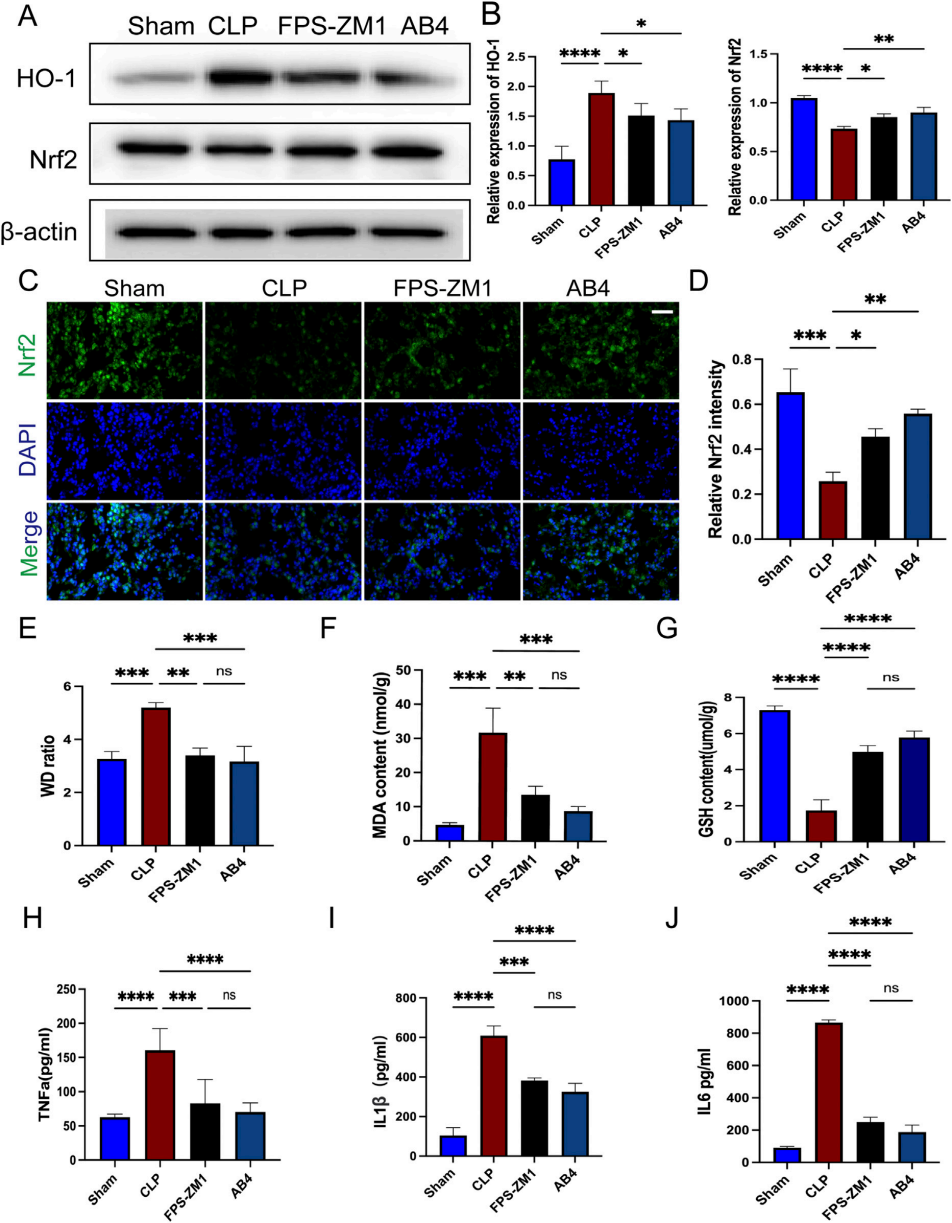
AB4 inhibit ferroptosis through the RAGE/Nrf2/HO-1signalling pathway. **(A,B)** Western blot analyses of Nrf2, HO-1. **(C,D)** Images of Nrf2 staining of lungs. Scale bar = 20 μm. Quantitative graph for RAGE staining of lungs (n = 3). **(E)** Determination of the wet/dry weight ratio of lung tissues in each group (n = 3). **(F,G)** The levels of GSH and MDA in the lungs (n = 3). **(H–J)** The TNF-α, IL-6, and IL-1β levels in mice serum (n = 3). Data are presented as means ± standard deviations, *p < 0.05, **p < 0.01, ***p < 0.001, ****p < 0.0001.

Western blot analysis showed that in the CLP group, Nrf2 expression was suppressed, whereas HO-1 expression was significantly upregulated (*p* < 0.001); both AB4 treatment and RAGE inhibition with FPS-ZM1 significantly reversed these trends (*p* < 0.05; Figures 10A,B). Additionally, increased nuclear translocation of Nrf2 was observed in the AB4 and FPS-ZM1-treated groups, indicating enhanced Nrf2 activity (*p* < 0.05; Figures 10C,D). Pathological assessment of lung injury demonstrated that compared with the CLP group, the lung wet/dry weight ratio was significantly reduced in both the FPS-ZM1 and AB4 treatment groups (*p* < 0.01; Figure 10E). Biochemical analyses revealed that the CLP group exhibited significantly elevated MDA levels and reduced GSH content (both *p* < 0.001). In contrast, both FPS-ZM1 and AB4 treatments effectively attenuated MDA accumulation (*p* < 0.01) and restored GSH concentrations (*p* < 0.001; Figures 10F,G). Detection of serum inflammatory factors revealed that compared with the CLP group, the levels of TNF-α, IL-1β, and IL-6 were significantly lower in both the FPS-ZM1 and AB4 groups (*p* < 0.01; Figures 10H–J), with no statistically significant difference between the two treatment groups. These results suggest that AB4 alleviates SALI by inhibiting the RAGE/Nrf2/HO-1 pathway, thereby reducing ferroptosis and inflammation.

## 4 Discussion

Sepsis is the leading cause of death in intensive care units, with SALI as a common complication often progressing to life-threatening multiple organ dysfunction (Zhang and Ning, 2021). Despite the high incidence and mortality (Duggal and Camporota, 2024), the pathogenesis of SALI remains incompletely understood, and effective treatments are limited. Ferroptosis—a distinct regulated cell death mechanism differing from apoptosis and autophagy—is characterized by Fe^2+^-dependent lipid peroxidation (Dixon et al., 2012). It plays significant roles in sepsis pathogenesis (Wu et al., 2024; Shen et al., 2023), with key molecular markers including PTGS2/COX2, SLC7A11 and GPX4 (Dixon et al., 2012; Yang et al., 2014; Lo et al., 2008). Accumulating studies indicate that active constituents derived from botanical drugs used in TCM exert beneficial pharmacological effects in SALI, including inflammation alleviation (Yuan et al., 2020), oxidative stress inhibition (Li et al., 2024), intestinal microbiota regulation (Bao et al., 2023), and modulation of cell death pathways (Li et al., 2024). As a key bioactive constituent of *Pulsatilla chinensis*, AB4 has emerged as a promising candidate for SALI therapy due to its multimodal mechanisms—specifically, its anti-inflammatory, antioxidant, and cell death regulatory properties. These multifaceted pharmacological effects align well with the complex pathophysiological characteristics of SALI, endowing it with unique application prospects.

Previous studies have reported that AB4 exerts protective pharmacological effects in SALI through mechanisms such as autophagy regulation via the mTOR/p70S6K pathway (Pei and He, 2021) and inhibition of the NLRP3 inflammasome (Yuan et al., 2020). While these mechanisms reduce inflammation, they fail to address the iron-dependent lipid peroxidation cascade, which can lead to irreversible alveolar damage. In the present study, AB4 improved lung pathological changes, reduced the levels of MDA, DHE and PTGS2, while increasing the expression of GSH, GPX4, and SLC7A11. Cysteine is crucial for GSH synthesis; GSH interacts with GPX4 to mediate redox reactions, converting toxic lipid hydroperoxides into non-toxic alcohols. Therefore, depletion of GSH and inhibition of GPX4 activity can induce ferroptosis (Carey et al., 2005). Additionally, AB4 not only inhibited ferroptosis but also simultaneously reduced the levels of proinflammatory cytokines (TNF-α, IL-6, and IL-1β). Further *in vitro* experiments using LPS-treated RAW264.7 cells demonstrated that AB4 dose-dependently decreased MDA levels, increased GSH content, upregulated the expression of GPX4 and SLC7A11, and suppressed the production of inflammatory cytokines (TNF-α, IL-6, and IL-1β), with pharmacological effects comparable to those of the ferroptosis inhibitor Fer-1. Collectively, these findings indicate that AB4 ameliorates SALI by inhibiting ferroptosis in lung tissues and macrophages.

**FIGURE 11:**
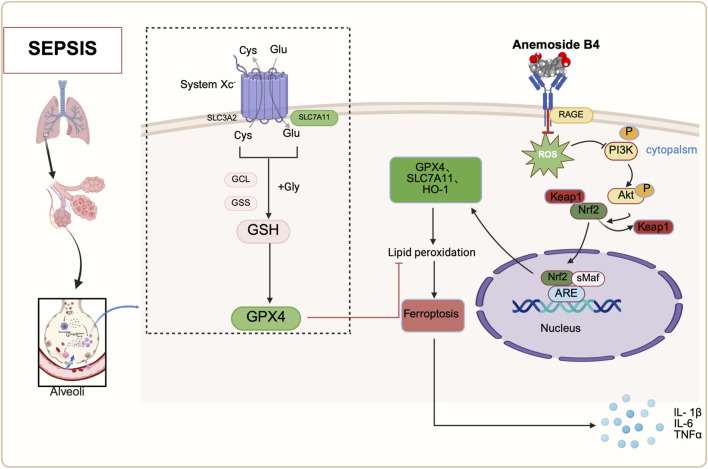
Schematic diagram of the mechanisms by which anemoside B4 (AB4) ameliorates SALI by regulating the AGE/RAGE/Nrf2 pathway. Adapted from BioRender [Created in BioRender. Chen, Y. (2025) https://BioRender.com/93y24c0], used under license. Copyright ^©^ [2025] BioRender. All rights reserved.

To clarify the protective mechanism by which AB4 inhibits ferroptosis in SALI, we integrated network pharmacology with experimental validation. GO and KEGG enrichment analyses were performed on the intersecting genes of AB4, acute lung injury (ALI), and ferroptosis. KEGG analysis identified the AGE/RAGE signaling pathway as the primary pathway involved. RAGE, a member of the immunoglobulin superfamily, is highly expressed on the surface of various cell types, with particularly high expression in lung tissue, and can bind to multiple ligands (He et al., 2023). In infectious contexts, ligands such as AGEs, S100 calpain, and high mobility group box 1 (HMGB1) bind to RAGE to initiate and amplify inflammatory response pathways (Buckley and Ehrhardt, 2010; Su et al., 2009). Elevated serum levels of RAGE ligands correlate with poor prognosis in viral pneumonia, including COVID-19 induced acute respiratory distress syndrome (Chen et al., 2020). RAGE acts as a mediator linking oxidative stress, inflammation, and immune responses (Pierine et al., 2014), and the RAGE ligand axis plays a key role in sustaining persistent inflammation (Creagh‐Brown et al., 2010). Furthermore, excessive activation of the AGE/RAGE signaling pathway exacerbates oxidative stress and mitochondrial dysfunction in sepsis (Cepas et al., 2020). Specifically, in SALI, this pathway acts as an upstream integrator of multiple injury drivers. Building on its known roles, pharmacological inhibition of RAGE has been shown to attenuate SALI (Xie et al., 2024; He et al., 2023). Critically, we now provide direct evidence that activation of the AGE/RAGE axis triggers ferroptosis—a key amplifier of oxidative damage in SALI. Given that ferroptosis is mechanistically linked to oxidative stress and mitochondrial failure (Stockwell et al., 2017), we hypothesized that AB4 may ameliorate SALI by targeting the AGE/RAGE-ferroptosis axis. Mechanistically, SPR analysis confirmed that AB4 directly binds to the RAGE V-domain with high affinity (KD = 3.86 μM), characterized by rapid association and slow dissociation kinetics. Molecular dynamics simulations further revealed stable binding mediated by key hydrogen bonds and hydrophobic interactions. Importantly, this RAGE engagement translated to functional pharmacological effects: in septic mice, AB4 dose-dependently suppressed activation of the AGE/RAGE signaling pathway and concurrently normalized ferroptosis markers (PTGS2, GPX4 and SLC7A11). These results demonstrate that AB4 inhibits ferroptosis by intercepting the AGE/RAGE signaling pathway, thereby alleviating SALI progression—consistent with reports that RAGE blockade attenuates ferroptosis (Chen et al., 2024; Zhou et al., 2023). The suppression of ferroptosis is further amplified through the rescue of Nrf2. RAGE activation promotes Nrf2 degradation (Wang et al., 2025), impairing its transcriptional regulation of ferroptosis-defense genes (SLC7A11 and GPX4) (Sun et al., 2016; Dong et al., 2021). Consistent with this mechanism, AB4 and the RAGE inhibitor FPS-ZM1 reversed Nrf2 depletion *in vivo*, restoring GPX4 and SLC7A11 expression. Consequently, glutathione metabolic flux was rescued (Figure 11), intercepting lipid peroxidation. Thus, Nrf2-driven antioxidant responses constitute a central effector mechanism downstream of RAGE, and the normalization of ferroptosis markers reflects the restoration of systemic redox homeostasis (Stockwell et al., 2017). Collectively, AB4 mitigates SALI by inhibiting the AGE/RAGE signaling pathway, thereby attenuating ferroptosis and inflammation. The concomitant modulation of Nrf2 suggests that a functional AGE/RAGE-Nrf2 axis contributes to this protective effect.

This study has certain limitations. Although the CLP model recapitulates relevant pathophysiological features, its systemic complexity necessitated complementary *in vitro* studies to dissect specific mechanisms. While our focus on macrophages—critical effector cells in SALI—yielded significant insights, experiments involving alveolar epithelial and endothelial cells are underway to further delineate AB4’s lung-specific actions. Additionally, while genetic validation (e.g., RAGE knockout models) was beyond the scope of the current work, pharmacological evidence (including synergistic effects with FPS-ZM1) and dose-response analyses provide robust support for our conclusions. Future studies will employ CRISPR-Cas9 technology to unequivocally establish RAGE dependency.

## 5 Conclusion

This study is the first to demonstrate that AB4 alleviates SALI by inhibiting ferroptosis through targeting the AGE/RAGE signaling pathway. Mechanistically, AB4 directly binds to the V-domain of RAGE (KD = 3.86 μM), thereby blocking AGE/RAGE signaling and activating Nrf2. This activation upregulates HO-1, GPX4, and SLC7A11, which collectively suppress ferroptosis, oxidative stress, and mitochondrial structural damage. Beyond its anti-ferroptotic role, AB4 exerts multimodal effects—particularly in combating inflammation—through this RAGE-Nrf2 axis. This unified mechanism offers a coordinated therapeutic strategy for sepsis-induced organ injury. Collectively, this work identifies AB4 as a promising candidate for SALI treatment through modulation of the AGE/RAGE-ferroptosis cascade, establishing a paradigm for multi-pathway intervention in sepsis therapy.

## Data Availability

The raw data supporting the conclusions of this article will be made available by the authors, without undue reservation.

## Abbreviations

SALI, Sepsis-induced acute lung injury; AB4, Anemoside B4; LPS, Lipopolysaccharide; CLP, Cecum ligation and puncture; PTGS2, Prostaglandin G/H synthase 2 (COX2); SLC7A11, Recombinant solute carrier family 7, member 11; GPX4, Glutathione peroxidase 4; ELISA, Enzyme-linked immunosorbent assay; GO, Gene Ontology; KEGG, Kyoto Encyclopedia of Genes and Genomes; ROS, Reactive oxygen species; DHE, Dihydroethidium; MDA, Malondialdehyde; GSH, Glutathione; AGE/RAGE, Advanced Glycation End Products/Receptor for Advanced Glycation End Products; HE, Hematoxylin and Eosin.

## References

[B1] Bao K. Wang M. Liu L. Zhang D. Jin C. Zhang J. (2023). Jinhong decoction protects sepsis-associated acute lung injury by reducing intestinal bacterial translocation and improving gut microbial homeostasis. Front. Pharmacol. 14, 1079482. 10.3389/fphar.2023.1079482 37081964 PMC10110981

[B2] Buckley S. T. Ehrhardt C. (2010). The receptor for advanced glycation end products (RAGE) and the lung. J. Biomed. Biotechnol. 2010, 917108. 10.1155/2010/917108 20145712 PMC2817378

[B3] Cao Z. Niu X. Wang M. Yu S. Wang M. Mu S. (2023). Anemoside B4 attenuates RANKL-induced osteoclastogenesis by upregulating Nrf2 and dampens ovariectomy-induced bone loss. Biomed. Pharmacother. 167, 115454. 10.1016/j.biopha.2023.115454 37688987

[B4] Carey M. A. Bradbury J. A. Seubert J. M. Langenbach R. Zeldin D. C. Germolec D. R. (2005). Contrasting effects of cyclooxygenase-1 (COX-1) and COX-2 deficiency on the host response to influenza A viral infection. J. Immunol. 175 (10), 6878–6884. 10.4049/jimmunol.175.10.6878 16272346

[B5] Cepas V. Collino M. Mayo J. C. Sainz R. M. (2020). Redox signaling and advanced glycation endproducts (AGEs) in diet-related diseases. Antioxidants 9 (2), 142. 10.3390/antiox9020142 32041293 PMC7070562

[B6] Chen L. Long X. Xu Q. Tan J. Wang G. Cao Y. (2020). Elevated serum levels of S100A8/A9 and HMGB1 at hospital admission are correlated with inferior clinical outcomes in COVID-19 patients. Cell. Mol. Immunol. 17 (9), 992–994. 10.1038/s41423-020-0492-x 32620787 PMC7332851

[B7] Chen X. Liu C. Yu R. Gan Z. Zhang Z. Chen Z. (2023). Interaction between ferroptosis and TNF-α: impact in obesity-related osteoporosis. FASEB J. 37 (6), e22947. 10.1096/fj.202201958R 37199646

[B8] Chen Y. Meng Z. Li Y. Liu S. Hu P. Luo E. (2024). Advanced glycation end products and reactive oxygen species: uncovering the potential role of ferroptosis in diabetic complications. Mol. Med. 30, 141. 10.1186/s10020-024-00905-9 39251935 PMC11385660

[B50] Creagh‐Brown B. C. Quinlan G. J. Evans T. W. Burke‐Gaffney A. (2010). The RAGE axis in systemic inflammation, acute lung injury and myocardial dysfunction: an important therapeutic target? Intensive Care Med. 36 (10), 1644–1656. 10.1007/s00134-010-1952-z 20631986

[B9] Dixon S. J. Lemberg K. M. Lamprecht M. R. Skouta R. Zaitsev E. M. Gleason C. E. (2012). Ferroptosis: an iron-dependent form of nonapoptotic cell death. Cell 149 (5), 1060–1072. 10.1016/j.cell.2012.03.042 22632970 PMC3367386

[B10] Dodson M. Castro-Portuguez R. Zhang D. D. (2019). NRF2 plays a critical role in mitigating lipid peroxidation and ferroptosis. Redox Biol. 23, 101107. 10.1016/j.redox.2019.101107 30692038 PMC6859567

[B49] Dong H. Xia Y. Jin S. Xue C. Wang Y. Hu R. (2021). Nrf2 attenuates ferroptosis-mediated IIR-ALI by modulating TERT and SLC7A11. Cell Death Dis. 12, 1027. 10.1038/s41419-021-04307-1 34716298 PMC8556385

[B11] Duggal A. Camporota L. (2024). Sequencing interventions in ARDS: the critical role of timing and order in standardized management. Intensive Care Med. 50, 1133–1136. 10.1007/s00134-024-07412-9 38695930

[B12] Fang J. Yuan Q. Du Z. Zhang Q. Yang L. Wang M. (2023). Overexpression of GPX4 attenuates cognitive dysfunction through inhibiting hippocampus ferroptosis and neuroinflammation after traumatic brain injury. Free Radic. Biol. Med. 204, 68–81. 10.1016/j.freeradbiomed.2023.04.014 37105419

[B13] Guo C. Yue Y. Wang B. Chen S. Li D. Zhen F. (2024). Anemoside B4 alleviates arthritis pain via suppressing ferroptosis-mediated inflammation. J. Cell. Mol. Med. 28, e18136. 10.1111/jcmm.18136 38334255 PMC10853948

[B14] He J. Yuan R. Cui X. Cui Y. Han S. Wang Q. Q. (2020). Anemoside B4 protects against klebsiella pneumoniae- and influenza virus FM1-induced pneumonia via the TLR4/Myd88 signaling pathway in mice. Chin. Med. 15, 68. 10.1186/s13020-020-00350-w 32625244 PMC7330533

[B15] He L. Zhang Y. Kang N. Wang Y. Zhang Z. Zha Z. (2019). Anemoside B4 attenuates nephrotoxicity of cisplatin without reducing anti-tumor activity of cisplatin. Phytomedicine 56, 136–146. 10.1016/j.phymed.2018.10.035 30668334

[B16] He R. Liu B. Xiong R. Geng B. Meng H. Lin W. (2022). Itaconate inhibits ferroptosis of macrophage via Nrf2 pathways against sepsis-induced acute lung injury. Cell death Discov. 8 (1), 43. 10.1038/s41420-021-00807-3 35110526 PMC8810876

[B17] He Y. Q. Deng J. L. Zhou C. C. Jiang S. G. Zhang F. Tao X. (2023). Ursodeoxycholic acid alleviates sepsis-induced lung injury by blocking PANoptosis via STING path way. Int. Immunopharmacol. 125 (Pt B), 111161. 10.1016/j.intimp.2023.111161 37948864

[B18] Huang F. Pang J. Xu L. Niu W. Zhang Y. Li S. (2022). Hedyotis diffusa injection induces ferroptosis via the Bax/Bcl2/VDAC2/3 axis in lung adenocarcinoma. Phytomedicine 104, 154319. 10.1016/j.phymed.2022.154319 35853302

[B19] Jin M. M. Zhang W. D. Song G. S. Xu Y. M. Du Y. F. Guo W. (2018). Discrimination and chemical phylogenetic study of four pulsatilla herbs using UPLC-ESI-MS/MS combined with hierarchical cluster analysis. J. Chromatogr. Sci. 56 (3), 216–224. 10.1093/chromsci/bmx102 29236950

[B20] Kang N. Shen W. Zhang Y. Su Z. Yang S. Liu Y. al. (2019). Anti-inflammatory and immune-modulatory properties of anemoside B4 isolated from Pulsatilla chinensis in vivo. Phytomedicine 64, 152934. 10.1016/j.phymed.2019.152934 31454651

[B21] Lai K. Song C. Gao M. Deng Y. Lu Z. Li N. (2023). Uridine alleviates sepsis-induced acute lung injury by inhibiting ferroptosis of macrophage. Int. J. Mol. Sci. 24 (6), 5093. 10.3390/ijms24065093 36982166 PMC10049139

[B22] Li H. Tang Z. Chu P. Song Y. Yang Y. Sun B. (2018). Neuroprotective effect of phosphocreatine on oxidative stress and mitochondrial dysfunction induced apoptosis in vitro and *in vivo:* involvement of dual PI3K/Akt and Nrf2/HO-1 pathways. Free Radic. Biol. Med. 120, 228–238. 10.1016/j.freeradbiomed.2018.03.014 29559323

[B23] Li J. Deng S. H. Li J. Li L. Zhang F. Zou Y. (2022). Obacunone alleviates ferroptosis during lipopolysaccharide-induced acute lung injury by upregulating Nrf2-dependent antioxidant responses. Cell. Mol. Biol. Lett. 27, 29. 10.1186/s11658-022-00318-8 35305560 PMC8933916

[B24] Li S. Li X. Yang R. Wang B. Li J. Cao L. (2019). Effects of anemoside B4 on pharmacokinetics of florfenicol and mRNA expression of CXR, MDR1, CYP3A37 and UGT1E in broilers. J. Vet. Med. Sci. 81 (12), 1804–1809. 10.1292/jvms.19-0293 31611492 PMC6943327

[B25] Li Y. H. Zou M. Han Q. Deng L. R. Weinshilboum R. M. (2020). Therapeutic potential of triterpenoid saponin anemoside B4 from Pulsatilla chinensis. Pharmacol. Res. 160, 105079. 10.1016/j.phrs.2020.105079 32679180

[B48] Li W Guo Y. Xu Z. Li F. Dong Y. Xu F. (2024). Notoginsenoside R1 (NGR1) regulates the AGE-RAGE signaling pathway by inhibiting RUNX2 expression to accelerate ferroptosis in breast cancer cells. Aging (Albany, NY). 16, 10446–10461. 10.18632/aging.205940 38885076 PMC11236304

[B26] Lo M. Ling V. Wang Y. Z. Gout P. W. (2008). The xc- cystine/glutamate antiporter: a mediator of pancreatic cancer growth with a role in drug resistance. Br. J. Cancer 99 (3), 464–472. 10.1038/sj.bjc.6604485 18648370 PMC2527809

[B27] Luo L. Huang F. Zhong S. Ding R. Su J. Li X. (2022). Astaxanthin attenuates ferroptosis via Keap1-Nrf2/HO-1 signaling pathways in LPS-induced acute lung injury. Life Sci. 311 (Pt A), 121091. 10.1016/j.lfs.2022.121091 36252699

[B28] Lutterloh E. C. Opal S. M. Pittman D. D. Keith J. C. Tan X. Y. Clancy B. M. (2007). Inhibition of the RAGE products increases survival in experimental models of severe sepsis and systemic infection. Crit. Care 11, R122. 10.1186/cc6184 18042296 PMC2246216

[B29] Ma H. Zhou Z. Chen L. Wang L. Muge Q. (2022). Anemoside B4 prevents chronic obstructive pulmonary disease through alleviating cigarette smoke-induced inflammatory response and airway epithelial hyperplasia. Phytomedicine 107, 154431. 10.1016/j.phymed.2022.154431 36115169

[B30] Malainou C. Abdin S. M. Lachmann N. Matt U. Herold S. (2023). Alveolar macrophages in tissue homeostasis, inflammation, and infection: evolving concepts of therapeutic targeting. J. Clin. Investig. 133 (19), e170501. 10.1172/JCI170501 37781922 PMC10541196

[B31] Matute-Bello G. Downey G. Moore B. B. Groshong S. D. Matthay M. A. Slutsky A. S. (2011). An official American thoracic society workshop report: features and measurements of experimental acute lung injury in animals. Cell. Mol. Biol. 44 (5), 725–738. 10.1165/rcmb.2009-0210ST PMC7328339 21531958

[B32] Pei L. He L. (2021). Hepatoprotective effect of anemoside B4 against sepsis-induced acute liver injury through modulating the mTOR/p70S6K-mediated autophagy. Chem. Biol. Interact. 345, 109534. 10.1016/j.cbi.2021.109534 34051206

[B33] Pierine D. T. Navarro M. E. Minatel I. O. Luvizotto R. A. M. Nascimento A. F. Ferreira A. L. A. (2014). Lycopene supplementation reduces TNF-α via RAGE in the kidney of obese rats. Nutr. Diabetes 4 (11), e142. 10.1038/nutd.2014.39 25383746 PMC4259904

[B34] Schindelin J. Arganda-Carreras I. Frise E. Kaynig V. Longair M. Pietzsch T. (2012). Fiji: an open-source platform for biological-image analysis. Nat. Methods 9 (7), 676–682. 10.1038/nmeth.2019 22743772 PMC3855844

[B35] Shen K. Wang X. Wang Y. Jia Y. Zhang Y. Wang K. (2023). miR-125b-5p in adipose derived stem cells exosome alleviates pulmonary microvascular endothelial cells ferroptosis via Keap1/Nrf2/GPX4 in sepsis lung injury. Redox Biol. 62, 102655. 10.1016/j.redox.2023.102655 36913799 PMC10023991

[B36] Stockwell B. R. Friedmann Angeli J. P. Bayir H. Bush A. I. Conrad M. Dixon S. (2017). Ferroptosis: a regulated cell death nexus linking metabolism, redox biology, and disease. Cell 171, 273–285. 10.1016/j.cell.2017.09.021 28985560 PMC5685180

[B37] Su X. Looney M. R. Gupta N. Matthay M. A. (2009). Receptor for advanced glycation end-products (RAGE) is an indicator of direct lung injury in models of experimental lung injury. Am. J. Physiol. Lung Cell. Mol. Physiol. 297 (1), L1–L5. 10.1152/ajplung.90546.2008 19411309 PMC2711813

[B38] Sun C.-Y. Yang L.-L. Zhao P. Yan P.-Z. Li J. Zhao D.-S. (2022). Mechanisms of cynarine for treatment of non-alcoholic fatty liver disease based on the integration of network pharmacology, molecular docking and cell experiment. Hereditas 159, 44. 10.1186/s41065-022-00256-7 36451177 PMC9714250

[B39] Sun X. Ou Z. Chen R. Niu X. Chen D. Kang R. (2016). Activation of the p62-Keap1 NRF2 pathway protects against ferroptosis in hepatocellular carcinoma cells. Hepatology 63, 173–184. 10.1002/hep.28251 26403645 PMC4688087

[B40] Wang J. Zhang Z. Yu M. Xin L. (2025). Cold stimulated bronchial epithelial cells derived exosomes HMGB1 aggravates bronchial epithelial cells injury. Mol. Immunol. 177, 96–103. 10.1016/j.molimm.2024.12.007 39755020

[B41] Wu D. Spencer C. B. Ortoga L. Zhang H. Miao C. (2024). Histone lactylation-regulated METTL3 promotes ferroptosis via m6A-modification on ACSL4 in sepsis-associated lung injury. Redox Biol. 74, 103194. 10.1016/j.redox.2024.103194 38852200 PMC11219935

[B42] Xie L. Zhang G. Wu Y. Hua Y. Ding W. Han X. (2024). Protective effects of wenqingyin on sepsis-induced acute lung injury through regulation of the receptor for advanced glycation end products pathway. Phytomedicine 129, 155654. 10.1016/j.phymed.2024.155654 38723525

[B44] Yang W. S. SriRamaratnam R. Welsch M. E. Shimada K. Skouta R. Viswanathan V. S. (2014). Regulation of ferroptotic cancer cell death by GPX4. Cell 156 (1–2), 317–331. 10.1016/j.cell.2013.12.010 24439385 PMC4076414

[B43] Yuan R. He J. Huang L. Du L. J. Gao H. Xu Q. (2020). Anemoside B4 protects against acute lung injury by attenuating inflammation through blocking NLRP3 inflammasome activation and TLR4 dimerization. J. Immunol. Res. 2020, 7502301. 10.1155/2020/7502301 33344657 PMC7732379

[B45] Zhang Y. Du X. He Z. Gao S. Ye L. Ji J. (2023). A vanadium-based nanoplatform synergizing ferroptotic-like therapy with glucose metabolism intervention for enhanced cancer cell death and antitumor immunity. ACS nano 17 (12), 11537–11556. 10.1021/acsnano.3c01527 37272777

[B46] Zhang Y. Y. Ning B. T. (2021). Signaling pathways and intervention therapies in sepsis. Signal Transduct. Target. Ther. 6 (1), 407. 10.1038/s41392-021-00816-9 34824200 PMC8613465

[B47] Zhou C. Yu T. Zhu R. Lu J. Ouyang X. Zhang Z. (2023). Timosaponin AIII promotes non-small-cell lung cancer ferroptosis through targeting and facilitating HSP90 mediated GPX4 ubiquitination and degradation. Int. J. Biol. Sci. 19 (5), 1471–1489. 10.7150/ijbs.77979 37056925 PMC10086754

