# Supplementary material for: Anemoside B4 targets RAGE to attenuate ferroptosis in sepsis-induced acute lung injury
Source: Front Pharmacol. 2025 Jul 31;16:1590797. doi: 10.3389/fphar.2025.1590797 (PMC12350364; doi:10.3389/fphar.2025.1590797)
Supplement: Supplementary file 2 [file Table3.docx]

**Table 3. T**abulating MM-GBSA results

|  | Mean ± SD | Contribution (%) |
| --- | --- | --- |
| Van der Waals | -50.2 ± 5.8 | 57.3% |
| Electrostatic | -30.1 ± 4.2 | 34.4% |
| Polar Solvation | 20.5 ± 3.5 | -23.4% |
| Non-polar Solvation | -15.6 ± 2.1 | 17.8% |
| Total Binding Energy | -43.41 ± 6.06 | 100% |
